# Evaluation of coagulation by thromboelastography and a velocity curve in dogs with parvoviral enteritis

**DOI:** 10.17221/49/2024-VETMED

**Published:** 2024-10-30

**Authors:** Oya Eralp Inan, Pinar Levent, Ahmet Saril, Lina Hamabe, Meric Kocaturk, Zeki Yilmaz

**Affiliations:** ^1^Department of Animal Science, Faculty of Agriculture, Eskisehir Osmangazi Univesity, Eskisehir, Turkey; ^2^Department of Veterinary Medicine, Facility of Agriculture, Tokyo University of Agriculture and Technology, Fuchu, Japan; ^3^Department of Internal Medicine, Faculty of Veterinary Medicine, Bursa Uludag University, Bursa, Turkey

**Keywords:** coagulation, fibrinolysis, hypercoagulation, hypocoagulation, inflammation, sepsis

## Abstract

Canine parvoviral enteritis (CPE) has a high mortality rate in untreated dogs due to systemic inflammation and multi-organ dysfunction. The inflammatory process can lead to coagulation abnormalities. This study aimed to evaluate the coagulation status using thromboelastography (TEG) and assess the thrombin generation (TG) and clot dissolution using TEG-derived velocity curve (v-curve) parameters in dogs with CPE. It included 21 dogs with CPE and five healthy dogs. In addition to the clinico-haemato-biochemical examinations, the coagulation status was analysed using citrated venous blood samples with TEG. All the dogs with CPE met at least two criteria for systemic inflammatory response syndrome (SIRS). The comparison to healthy controls showed a statistically significant prolongation of reaction times (R time; *P* = 0.005) and times to the maximum rate of thrombus generation (TMRTG; *P* = 0.003). However, the times to the maximum rate of lysis (TMRL; *P* = 0.041) and total lysis (L; *P* = 0.024) decreased significantly. The TEG tracings showed coagulation states varying from hypocoagulation to hypercoagulation in dogs with CPE. These results showed that the v-curve derivate can be used to evaluate the coagulation in dogs with CPE, and it could be superior to the standard TEG variables for determining the low fibrinolytic activity. Thus, the v-curve parameters may provide a novel insight into the underlying mechanism and clinical treatment strategy of CPE-induced inflammation.

Canine parvoviral enteritis (CPE) is an enteropathogenic viral disease with high morbidity and mortality in young dogs (Alves et al. 2020). Canine parvovirus type 2 (CPV-2), the most common causative agent, targets rapidly dividing cells of the intestine, bone marrow and myocardium, resulting in vomiting, diarrhoea, leucopoenia, immunosuppression and myocarditis (Mazzaferro 2020; Corda et al. 2023). Altered microbiota, small intestinal bacterial overgrowth and bacterial invasions, and endotoxin propagation from the intestine to the systemic circulation led to systemic inflammatory response syndrome (SIRS) and sepsis in dogs with CPE (Otto et al. 1997; Kocaturk et al. 2012; Corda et al. 2023).

SIRS triggers coagulation in several ways (Stokol 2022). The increasing serum concentrations of acute-phase proteins in dogs with CPE reportedly have important roles in the development of multiple organ failure and coagulation abnormalities such as disseminated intravascular coagulation – DIC (Kocaturk et al. 2010; Alves et al. 2020). Proinflammatory cytokines stimulate a tissue factor release from injured endothelial cells and monocytes, which activates hypercoagulation followed by hypocoagulation and DIC (Donahue and Otto 2005). In dogs with CPE, the loss or excessive use of natural anticoagulants, such as antithrombin III (ATIII), makes them suitable for DIC or consumptive coagulopathy (Corda et al. 2023).

In human and veterinary medicine, in the diagnosis of coagulopathy, several indictors are measured, such as the platelet count and function, coagulation factors, natural anticoagulants (ATIII and proteins S and C), thrombin generation (TG) and fibrin split products (d-dimer). Among these, TG is considered better than conventional global tests, such as the prothrombin time (PT) and activated partial thromboplastin time (aPTT), for investigating hypo- and hypercoagulability (Tafur et al. 2016). The standard procedure for a TG analysis includes the use of platelet-poor plasma and the standard coagulation tests, PT and aPTT, better indicate the bleeding tendency. Therefore, these methods are unsuitable or insufficient for the routine analysis of haemostasis (Eralp Inan et al. 2016; Tafur et al. 2016; Yilmaz et al. 2017; Kim et al. 2020; Stokol 2022; Kocaturk et al. 2023).

Thromboelastography (TEG) and velocity curve (v-curve) derivatives have the advantages of being easy to use and giving details on the coagulation steps from the initiation of the clot formation to clot dissolution [fibrinolysis (Tafur et al. 2016)] which make them more popular in human (Kim et al. 2020; Mohan et al. 2022) and veterinary medicine (Eralp Inan et al. 2016; Yilmaz et al. 2017; Kocaturk et al. 2023; Rank et al. 2023). Previous studies have evaluated coagulation abnormalities using standard TEG [reaction (R) time, kinetic (K) time, maximum amplitude (MA), and alpha (α) angle] and traditional coagulation parameters in CPE, and their results were found to be compatible with a hypercoagulable state (Otto et al. 2000; Whitehead et al. 2020; Corda et al. 2023). The v-curve derivatives from TEG provide further details about the coagulation cascade through assessments of TG and clot dissolution (Engelen et al. 2017; Valke et al. 2022). The v-curve parameters evaluate clot formation by the maximum TG (MRTG) rate, time to maximum TG rate (TMRTG), and TG values. Clot dissolution is assessed by the maximum rate of lysis (MRL), time to maximum rate of lysis (TMRL) and total lysis (L). However, no study has investigated the coagulation process from TG to clot dissolution using TEG in dogs with CPE. Therefore, this study aimed to assess the coagulation status using TEG and v-curve data, as well as to evaluate whether a correlation existed between the two variables in dogs with CPE, a model of naturally occurring sepsis.

## MATERIAL AND METHODS

### Dogs

The dogs in this study were selected from previous studies of dogs with CPE complicated with sepsis (Kocaturk et al. 2012; Kocaturk et al. 2015). Twenty-one client-owned dogs of five different breeds (13 males and eight females, 3–17.8 kg in body weight, and less than six months of age) were included. The major inclusion criteria were that the dogs were untreated before admission to the clinic and had a positive test result for a faecal CPE antigen (Antigen Rapid CPV Kit; Animal Genetics, Inc., Suwon, Republic of Korea). Additionally, all the dogs showed clinical symptoms of vomiting and diarrhoea 1–3 days before presentation to the Bursa Uludag University Animal Teaching Hospital (Bursa, Türkiye). The dogs were identified as septic if serological evidence of an infection existed and they met at least two of four SIRS criteria: hypothermia or fever (temperature ≤ 37.8 °C or ≥ 39.7 °C, respectively), tachypnoea (respiratory rate ≥ 40 breath/min), tachycardia (heart rate ≥ 160 bpm), or leucopoenia (WBC count ≤ 4 000/μl) or leucocytosis (WBC count ≥ 12 000/μl), as previously described (Okano et al. 2002; Yilmaz and Senturk 2007; Kocaturk et al. 2010; Kocaturk et al. 2012; Corda et al. 2019). In these dogs, systemic inflammation was confirmed by the increasing serum levels of the C-reactive protein (CRP), a non-specific inflammatory marker for dogs (Kocaturk et al. 2010; Kocaturk et al. 2012).

The control group consisted of five dogs of four different breeds (three males and two females, 1–6 months old, and 8–23 kg in weight) appearing healthy based on the physical, haematological and biochemical examinations. After CPE was diagnosed, venous blood samples were collected, and the TEG and v-cure analyses were performed at room temperature within one hour after the sampling. These dogs were monitored during treatment based on the clinical and haemato-biochemistry analysis, but the TEG analysis was performed once before the treatment began.

### Thromboelastography

Venous blood samples (2 ml) were collected directly into vacuum tubes containing a 3.18% trisodium citrate anticoagulant with an 18-gauge non-heparinised needle from the brachiocephalic vein of all the dogs. A kaolin-activated TEG analysis was performed as a single test with recalcified citrated whole blood according to the manufacturer’s recommendations, using a TEG^®^ 5000 analyser [Haemonetics Corporation, Braintree, MA, USA (Bauer et al. 2009)].

The standard TEG parameters were the R time, K time, MA, α-angle, G value, coagulation index (CI) and lysis time (LY30). The v-curve derivates from TEG were the parameters of the clot formation (MRTG, TMRTG and TG) and clot dissolution (MRL, TMRL and L).

### Statistical analysis

In the comparison of the median differences between the two groups (healthy dogs vs dogs with CPE), the Mann–Whitney *U* test was used with a 95% confidence interval (1-α). The analysis of all the variables used R software (“base” package; Team RC 2022). *P*-values of less than 0.05 or 0.01 were considered statistically significant.

### Ethical approval

This study was approved by the Ethics and Welfare Committee of Bursa Uludag University (2010-06/10; 24.08.2010; Bursa, Türkiye).

## RESULTS

The dogs with CPE fulfilling the SIRS criteria represented both sexes (13 males, 61.9%; 8 females, 29.1%) and five different breeds (five crossbreeds, 23.8%; five Labrador Retrievers, 23.8%; five Kangal Shepherds, 23.8%; three Rottweilers, 14.2%; and three Pointers, 14.2%). Thirteen dogs (62%) met at least two of the SIRS criteria, and eight (38%) met three criteria. The control group consisted of both sexes (three males, 60%; two females, 40%) and four different breeds (two crossbreeds, 40%; one Rottweiler, 20%; one Labrador Retriever, 20%; and one Kangal Shepherd, 20%). The median values of the age and body weight in the CPE and control groups were four months and 11.8 kg, and four months and 12.0 kg, respectively. No significant differences existed in the age (*P* = 0.753) or body weight (*P* = 0.569) between the groups (Table 1). Selected clinical, haematological and serum biochemistry findings from the dogs are given in Table 1.

**Table 1:** Some signalment, haematological and CRP variables of the control dogs and dogs with CPE

Variable	Control (*n* = 5)			CPE (*n* = 21)		*P*-value
	median	IQR		median	IQR	
Age (months)	4.00	2.50–5.00		4.00	3.00–5.00	0.753
Body weight (kg)	12.00	9.00–19.00		11.80	7.00–16.00	0.569
Heartbeat (bpm)	112.00	102.00–140.00		116.00	90.00–150.00	1.000
Respiratory rate (breaths/min)	28.00	25.00–36.00		36.00	30.00–40.00	0.138
Body temperature (°C)	38.30	38.15–38.50		38.90	38.35–39.40	0.079
Capillary refilling time (sec)	1.00^b^	1.00–2.00		2.00^a^	2.00–3.00	0.012*
WBC (×10^9^/l)	12.97^A^	11.16–21.69		3.49^B^	2.48–5.61	0.000**
HCT (%)	36.17^b^	30.27–40.27		41.53^a^	39.35–45.64	0.049*
PLT (×10^9^/l)	230.00	210.50–331.00		306.00	235.50–430.00	0.224
CRP (mg/l)	8.00^A^	4.30–15.15		98.10^B^	93.95–102.50	0.001**

*P < 0.05 (^a,b^); **P < 0.01 (^A,B^)

CRP = C-reactive protein; Hct = haematocrit; IQR = interquartile range (25^th^–75^th^ percentiles); PLT = platelet count; WBC = white blood cell count

Briefly, dogs with sepsis due to CPE showed some clinical and haematological abnormalities including pyrexia, increased heart and respiratory rates, a prolonged capillary refilling time, haemoconcentration, leucopoenia, and increased hepatorenal and cardiomyocyte injury markers compared to the reference values (Villiers et al. 2016). However, when the parameters in the CPE group were compared to those in the control group, the number of statistically significant variables decreased [see Electronic Supplementary Material (ESM) Table S1 for the haematology and Table S2 for the routine serum biochemistry panel].

**Figure 1:**
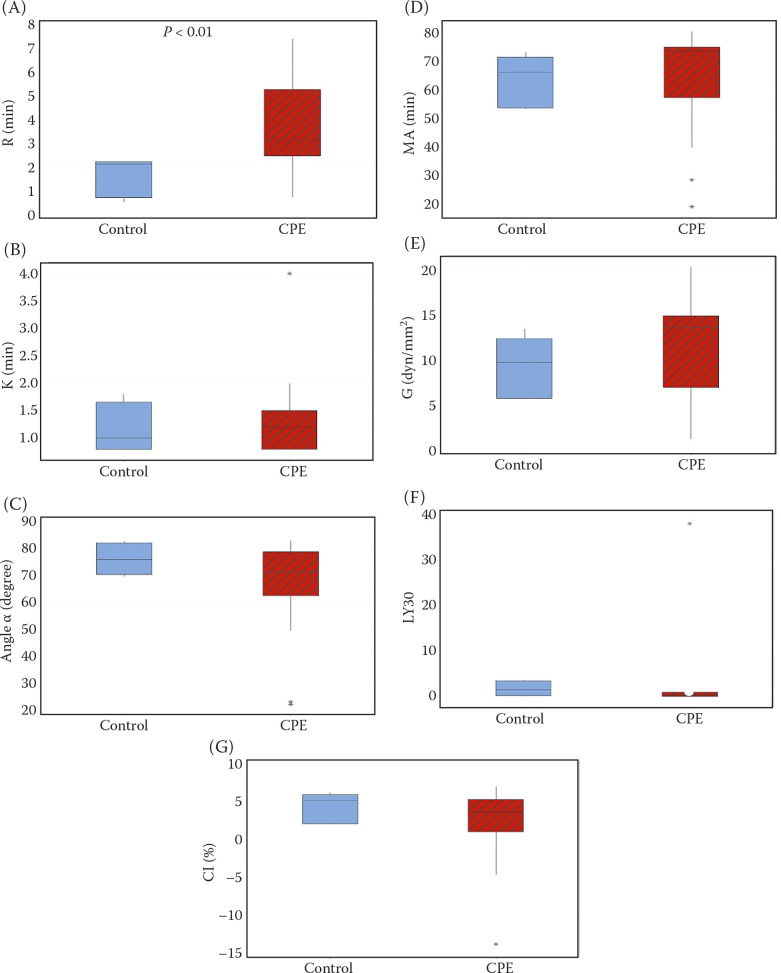
Traditional TEG variables expressed as box diagrams in the healthy and dogs with CPE The box represents the values from the lower to upper percentile and the middle line represents the median. (A) R = reaction time; (B) K = coagulation time; (C) Angle = α-angle, (D) MA = maximum amplitude; (E) G = clot rigidity; (F) LY30 = lysis at 30 min; (G) CI = coagulation index *Significance was set at *P* < 0.01

**Figure 2:**
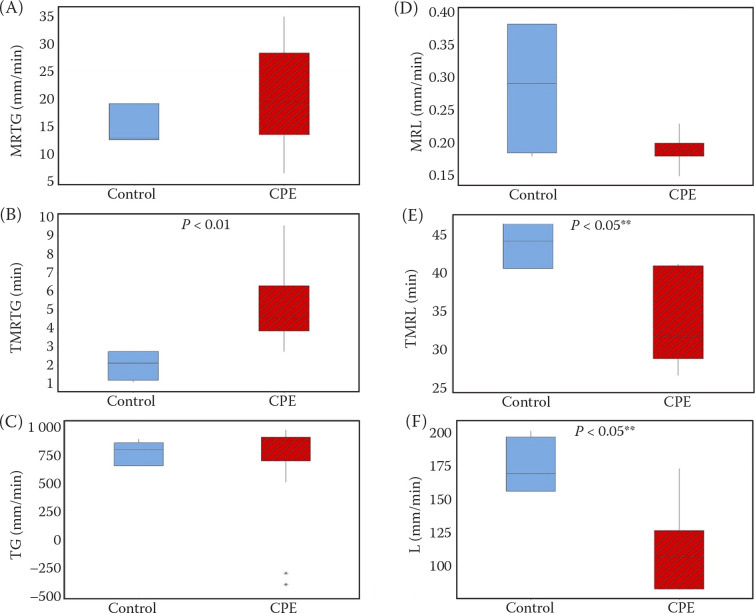
TEG-derived v-curve variables expressed as box diagrams in the healthy and dogs with CPE The box represents the values from the lower to upper percentile and the middle line represents the median. (A) MRTG = maximum rate of the thrombus generation; (B) TMRTG = time to the maximum rate of the thrombus generation; (C) TG = thrombus generation; (D) MRL = maximum rate of lysis; (E) TMRL = time to the maximum rate of lysis; (F) L = total lysis *Significance was set at *P* < 0.01; **Significance was set at *P* < 0.05

The results of the TEG analysis are shown in Table 2 and Figures 1 and 2. Some TEG variables showed statistically significant changes between the dogs with CPE and the healthy controls. The TEG R time and TMRTG increased (*P* < 0.01 and *P* < 0.05; respectively), whereas the TMRL and L values decreased (*P* < 0.05), in the dogs with CPE compared to the healthy controls. No statistically significant changes existed in the parameters of the K time, α-angle, MA, G, CI, LY30 or TG between the groups. On the other hand, the MA, G, MRTG and TG tended to increase, whereas MRL tended to decrease, in the dogs with CPE compared to the healthy ones.

**Table 2:** TEG variables in the control dogs and dogs with CPE

Variable	Control (*n* = 5)			CPE (*n* = 21)		*P*-value
	median	IQR		median	IQR	
R (min)	2.10^B^	0.70–2.20		3.10^A^	2.45–5.25	0.005**
K (min)	1.00	0.80–1.65		1.20	0.80–1.50	0.594
Angle (degree°)	75.70	70.20–81.80		71.30	62.40–78.55	0.256
MA (min)	65.90	53.45–71.20		73.30	57.10–74.80	0.170
G (dyn/cm^2^)	9.70	5.75–12.40		13.70	6.95–14.90	0.170
CI (%)	5.10	2.10–5.90		3.65	1.02–5.27	0.591
LY30	1.40	0.10–3.35		0.30	0.00–0.90	0.155
MRTG (mm/min)	12.81	12.55–19.10		19.41	13.47–28.28	0.192
TMRTG (min)	2.13^B^	1.19–2.75		4.55^A^	3.85–6.31	0.003**
TG (mm/min)	789.20	648.10–852.10		884.00	690.00–903.00	0.224
MRL (mm/min)	0.29	0.19–0.38		0.19	0.18–0.20	0.158
TMRL (min)	44.33^a^	40.75–46.50		31.88^b^	29.02–41.10	0.041*
L (mm/min)	169.50^a^	156.10–196.40		107.90^b^	83.70–127.10	0.024*

*P < 0.05 (^a,b^); **P < 0.01 (^A,B^)

Angle = α-angle; CI = coagulation index; CPE = canine parvoviral enteritis; G = clot rigidity; IQR = interquartile range (25^th^–75^th^ percentiles); K = coagulation time; L = total lysis; LY30 = lysis at 30 min; MA = maximum amplitude; MRL = maximum rate of lysis; MRTG = maximum rate of thrombus generation; R = reaction time; TEG = thromboelastography; TG = thrombus generation; TMRL = time to maximum rate of lysis

**Figure 3:**
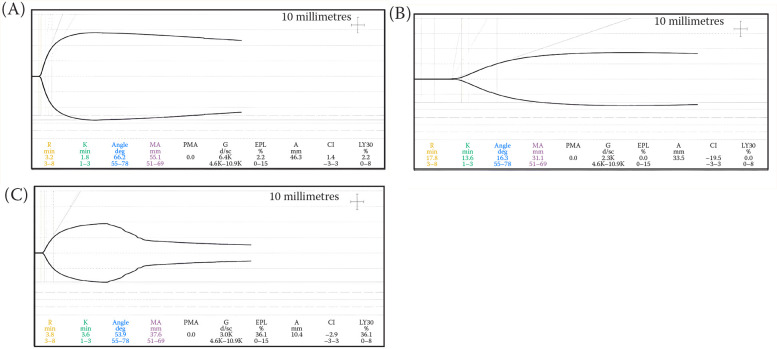
Thromboelastography tracings (A) Normocoagulable TEG tracing healthy dog; (B) Hypocoagulable TEG tracing in a dog with CPE; (C) Primary fibrinolysis in a dog with CPE

**Figure 4:**
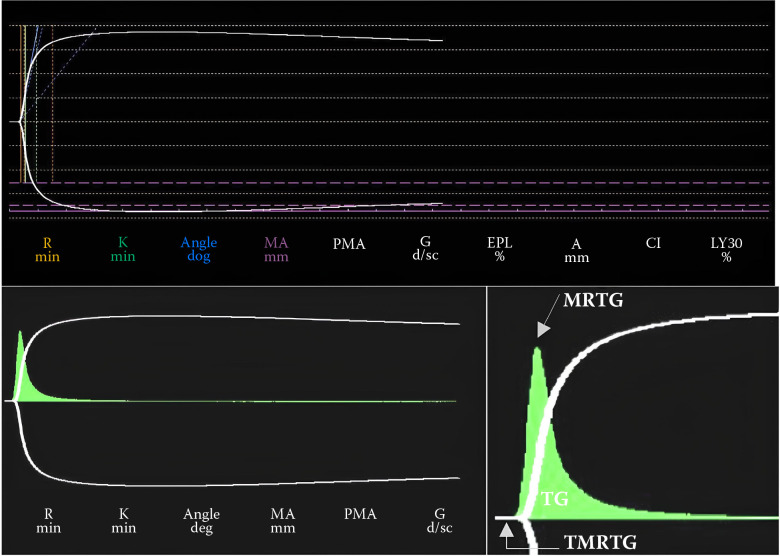
Thromboelastographic analysis (white colour) of a 4-month-old mixed breed dog with CPE (up) and velocity curve analysis (green colour) of the same thromboelastographic analysis (down). Hypercoagulable states are seen on both results MRTG = maximum rate of the thrombus generation; TG = total thrombus generation; TMRTG = time to the maximum rate of the thrombus generation

Figure 3 represents TEG tracings of the normo- and hypocoagulable states and primary fibrinolysis in dogs with CPE. Among the dogs with CPE, one fulfilled the hypercoagulation criteria, and another had primary fibrinolysis. Figure 4 shows the hypercoagulable TEG tracing and v-curve variables of a dog with CPE.

## DISCUSSION

This study showed, for the first time, the changes in the coagulation status determined by the v-curve derivatives from a TEG analysis in dogs with CPE, as a model of sepsis. Our results are compatible with the superiority of the v-curve data over traditional TEG measurements for assessing TG and fibrinolysis in dogs with sepsis due to CPE. TEG combined with v-curve data could be used as a fast and accurate bedside diagnostic tool to determine the coagulation status in clinical settings.

Coagulation abnormalities in sepsis range from hypocoagulation to hypercoagulation and various fibrinolytic disorders (Zhou et al. 2019). The results of this study showed a significant prolongation in the TG time and impaired fibrinolysis based on the v-curve evaluations in dogs with CPE. In contrast, a previous study establishing TEG variables in dogs with CPE showed hypercoagulative states in all dogs, based on the high MA and low ATIII levels (Otto et al. 2000). However, in our study, only one dog with CPE had a hypercoagulative state with a high TG result (Figure 4). This may be related to differences in the coagulation status in response to different severities of CPE.

Prolonged R times in dogs with CPE were consistent with a recently published study reporting an elevated aPTT (Corda et. 2023). However, in contrast to the results of another study (Whitehead et al. 2020), higher K and lower α-angle values were not observed. The R, K and α-angle variables measure the time to and rate of clot generation (Donahue and Otto 2005). The MA and its exponential reflection G evaluations showed no statistically significant alterations, but remained higher compared to the controls, indicating enhanced clot rigidity due to hypercoagulation (Muller et al. 2014). The R time is primarily influenced by plasma clotting factors, natural anticoagulants and coagulation inhibitors (Bauer et al. 2009; Engelen et al. 2017). The hypocoagulative status is generally identified by a high R and K or low MA and α-angle (Muller et al. 2014), as described in one dog with CPE in this study (Figure 3B). Additionally, the changes in the α-angle were closer to hypocoagulability.

The haemoconcentration (dehydration) may be a reason for the R prolongations in this study, in agreement with the results of a previous study indicating a positive correlation between the R time and Hct value in dogs with CPE (Whitehead et al. 2020). A prolonged R time may also result from low levels of coagulation factors as a consequence of dehydration [loss of plasma volume and proteins (Whitehead et al. 2020)]. Excessive use of coagulation proteins such as factors V, VII, VIII and X during hypercoagulative states in dogs with CPE can prolong the R time, suggestive of consumptive coagulopathy or DIC (Otto et al. 2000; Whitehead et al. 2020; Stokol 2022; Corda et al. 2023). The K value appeared to be unaffected in this study. It is influenced by the coagulation factors, fibrinogen, platelet count and Hct value (Donahue and Otto 2005). Factors, such as a high Hct that affect the R value, did not appear to affect the K time in this study. Fibrinogen, one of the possible factors modulating coagulation, may affect the K result since no statistically significant difference was observed in the platelet counts between the groups. However, in a prior study, high fibrinogen levels in CPE dogs did not shorten the K time as expected (Whitehead et al. 2020).

On the other hand, the v-curve-derived TMRTG value reflecting the clot formation was significantly higher in dogs with CPE compared to the controls in this study. This may be associated with the prolongation of the R time rather than the TG value as the major component of TMRTG (Gonzalez et al. 2010; Engelen et al. 2017) since the TG values in dogs with CPE did not show statistically significant differences compared to the control group. The TMRTG seemed to be affected, rather than the total TG and MRTG values, in dogs with CPE, similar to the results of the v-curve parameters in cats (Engelen et al. 2017). The TMRTG value is linearly related to the R time and TEG delta value in terms of TG (Engelen et al. 2017). Unfortunately, the delta value could not be evaluated in this study because its measurement was not available technically in the TEG used.

Decreased ATIII activity has been shown in not only dogs with CPE (Otto et al. 2000; Corda et al. 2023), but also dogs with protein-losing enteropathy (Goodwin et al. 2011) and in those having nephrotic syndrome (Lennon et al. 2013). The inflammation-based release of cytokines and the overexpression of the tissue factor could result from the consumption of natural anticoagulants such as active protein C, ATIII and tissue factor pathway inhibitors (Clavijo et al. 2019; Stokol 2022). Natural anticoagulants are greatly important in modulating the coagulation process, providing the balance between fibrin formation (coagulation) and fibrin dissolution [fibrinolysis (Zhou et al. 2019)]. In this study, the fibrinolytic system was evaluated by TEG v-curve parameters such as TMRL, MRL and L, but not others such as fibrin degradation products (FDPs), d-dimer or plasminogen. The v-curve variables, TMRL and L, were significantly lower, with a tendency to decrease in the MRL value, compared to those of the controls. Furthermore, the observed (but not statistically significant) increases in the MA, G and TG values in dogs with CPE seem to support a decrease in the fibrinolytic system.

One dog in this study had primary hyperfibrinolysis (Figure 3C). However, hypofibrinolytic activity was reported in dogs with underlying diseases with a high risk of thrombotic disorders (Spodsberg et al. 2013). The hypofibrinolytic state is considered a factor for thrombotic events (Kupesiz et al. 2010). Considering that dogs with sepsis were included in this study, the increase in the thrombin-activated fibrinolysis inhibitor activity may be another factor in the decreasing fibrinolysis (Jesses et al. 2010). Although the standard TEG variables showed no changes regarding the fibrinolysis, the v-curve results indicated hypofibrinolysis, indicating that v-curve variables could make an important contribution to evaluating coagulation in dogs with CPE.

A hypercoagulable state was not defined based on our TEG TG value in dogs with CPE, except in one case. A decreased TG is associated with a bleeding tendency, but an exaggerated TG is associated with an increased thrombotic risk (Binder et al. 2021). Thus, the observed increase in the TG value may be used as an indicator of thrombotic risk in dogs with CPE. Furthermore, previous studies have found differences between the results of the coagulation parameters. The d-dimer results in the studies of Otto et al. (2000) and Corda et al. (2023) were incompatible. Inconsistencies in the TEG results exist between our study and others (Otto et al. 2000). This may result from different sample sizes or disease severities. However, our results also showed that individual coagulation abnormalities, such as hypercoagulation, hypocoagulation and primary fibrinolysis, could not represent the overall coagulation status of dogs with CPE. This study provides the first data showing the hypofibrinolytic activity based on TEG v-curve values in dogs with CPE.

The study has several limitations. One is the lack of some coagulation parameters, such as the TEG delta, serum ATIII and d-dimer levels. Parameters reflecting fibrinolysis could have been a good contribution to assess their relationship with the v-curve fibrinolysis data. The use of TEG in veterinary medicine has been demonstrated by various studies; however, in practice, it has limitations due to high costs and the necessity of access to the device within 1–2 hours (Corda et al. 2023). On the other hand, TEG could be used as a fast and accurate bedside diagnostic tool for determining the coagulation status in clinical settings (Bauer et al. 2009)*.* The absence of blood gas measurements prevented us from obtaining detailed information about the metabolic status. Undetected diseases and the unknown vaccination status of the dogs could have been other limitations. If the clinical outcome of patients could be monitored and thromboembolic disorders could be diagnosed by histopathology or necropsy, the diagnostic importance of TEG parameters in dogs with CPE could be better emphasised.

In conclusion, the dogs with CPE in our study had downregulated fibrinolysis based on the TEG v-curve variables, which were not detectable with basic TEG measurements. Therefore, v-curve derivatives should be used along with standard TEG measurements. This can increase the coagulation status diagnostic accuracy and help in making true decisions for the medical management of dogs with CPE, as a model of sepsis.
