# Supplementary material for: Evaluation of coagulation by thromboelastography and a velocity curve in dogs with parvoviral enteritis
Source: Vet Med (Praha). 2024 Oct 30;69(10):345–54. doi: 10.17221/49/2024-VETMED (PMC11581469; doi:10.17221/49/2024-VETMED)
Supplement: Supplementary Tables 1 [file VETMED-69-10-124049-s001.pdf]

https://doi.org/10.17221/49/2024-VETMED

Evaluation of coagulation by thromboelastography and a velocity curve in dogs with parvoviral enteritis

Oya Eralp Inan[1]*, *Pinar Levent*[3], *Ahmet Saril*[3], *Lina Hamabe*[2], *Meric Kocaturk*[3], *Zeki Yilmaz*[3]

[1]*Department of Animal Science, Faculty of Agriculture, Eskisehir Osmangazi Univesity, Eskisehir, Turkey*

[2]*Department of Veterinary Medicine, Facility of Agriculture, Tokyo University of Agriculture and Technology, Fuchu, Japan*

[3]*Department of Internal Medicine, Faculty of Veterinary Medicine, Bursa Uludag University, Bursa, Turkey*

**Corresponding author: oeralp@ogu.edu.tr*

The authors are fully responsible for both the content and the formal aspects of the electronic supplementary material. No editorial adjustments were made.

Electronic Supplementary Material (ESM)

Table S1. Haematological findings in control dogs and dogs with CPE

Table S2. Serum biochemistry and cTnI results in control dogs and dogs with CPE

https://doi.org/10.17221/49/2024-VETMED

Table S1. Haematological findings in control dogs and dogs with CPE

Variable	Control (n = 5)		CPE (n = 21)		P-value
	median	IQR	median	IQR	
WBC (×10^9/l)	12.97^A	11.16–21.69	3.49^B	2.48–5.61	0.000**
LYM (×10^9/l)	3.26^A	2.55–3.78	0.97^B	0.65–1.27	0.000**
MONO (×10^9/l)	0.54^A	0.30–0.60	0.18^B	0.12–0.29	0.008**
NEUT (×10^9/l)	8.71^A	7.31–17.34	2.01^B	1.30–4.11	0.001**
EOS (×10^9/l)	0.29^A	0.25–0.54	0.06^B	0.04–0.09	0.001**
BASO (×10^9/l)	0.11^A	0.09–0.13	0.02^B	0.01–0.04	0.000**
RBC (×10^{12}/l)	5.62^B	5.05–6.06	6.82^A	5.91–7.40	0.006**
HGB (g/l)	11.50	9.80–13.05	13.60	12.30–14.45	0.067
HCT (%)	36.17^b	30.27–40.27	41.53^a	39.35–45.64	0.049*
MCV (fL)	62.00	60.00–67.50	61.00	58.00–66.00	0.409
MCH (%)	19.70	19.40–21.90	19.20	18.65–20.70	0.157
MCHC (g/l)	32.00	31.75–32.80	32.30	30.65–32.75	0.613
RDWC (%)	16.00^b	15.40–16.25	17.10^a	16.05–18.15	0.028*
PLT (×10^9/l)	230.00	210.50–331.00	306.00	235.50–430.00	0.224
PCT (%)	0.22	0.19–0.30	0.26	0.20–0.36	0.569
MPV (fL)	9.60	8.70–47.40	8.55	7.53–9.63	0.169
PDWC (%)	41.00	38.55–42.95	39.80	35.80–42.15	0.278

*P < 0.05 (^{a,b}); **P < 0.01 (^{A,B})

BASO = basophils; EOS = eosinophils; HCT = haematocrit; HGB = haemoglobin; IQR = interquartile range (25th–75th percentiles); MCH = mean corpuscular haemoglobin; MCHC= mean corpuscular haemoglobin concentration; MCV = mean corpusculum volume; MONO = monocytes; MPV = mean platelet volume; NEUT = neutrophils; PCT = plateletcrit; PDWC = platelet distribution width; PLT = platelet; RBC = erythrocytes; RDWC = red cell distribution width; WBC = white blood cell count

https://doi.org/10.17221/49/2024-VETMED

Table S2. Serum biochemistry and cTnI results in control dogs and dogs with CPE

Variable	Control (n = 5)		CPE (n = 21)		P-value
	median	IQR	median	IQR	
ALB (g/l)	3.40	2.48–3.65	2.90	2.55–3.10	0.154
ALP (U/l)	117.00	53.50–152.00	145.00	115.00–210.00	0.231
ALT (U/l)	24.00	22.30–54.30	38.00	22.00–56.50	0.642
AMY (U/l)	482.00	298.30–574.30	517.00	370.50–720.50	0.642
TBIL (μmol/l)	0.20[B]	0.20–0.20	0.30[A]	0.30–0.40	0.004**
BUN (mmol/l)	14.00	6.00–22.00	12.00	9.50–18.50	0.858
CA (mmol/l)	10.55	8.25–11.50	10.30	10.00–10.70	0.642
PHOS (mmol/l)	6.50	4.03–10.17	8.00	7.43–9.53	0.183
CREA (μmol/l)	0.60	0.35–0.78	0.60	0.30–0.70	0.748
GLU (mmol/l)	92.00	81.50–113.75	121.00	95.50–143.50	0.203
NA (mmol/l)	135.00	131.00–139.00	132.00	130.50–134.50	0.295
K (mmol/l)	5.00[a]	4.93–5.68	4.50[b]	4.05–4.65	0.011*
TP (g/l)	5.20	4.35–6.50	5.10	4.55–5.80	0.971
GLOB (g/l)	1.90	1.65–3.05	2.50	2.00–2.70	0.369
cTnI (ng/l)	0.07	0.07–0.12	0.01	0.00–0.08	0.167

*$P < 0.05$ ([a,b]); **$P < 0.01$ ([A,B])

ALB = albumin; ALP = alkaline phosphatase; ALT = alanine transaminase; AMY = amylase; BUN = blood urea nitrogen; CA = calcium; CREA = creatinine; cTnI = cardiac troponin I; GLOB = globulin; GLU = glucose; IQR = interquartile range (25th–75th percentiles); K = potassium; NA = sodium; PHOS = phosphate; TBIL= total bilirubin; TP = total protein
